# Ultrasonic Propagation in Liquid and Ice Water Drops. Effect of Porosity

**DOI:** 10.3390/s21144790

**Published:** 2021-07-13

**Authors:** Michiel Mendonck, Sofía Aparicio, Cristóbal González Díaz, Margarita G. Hernández, Guillermo M. Muñoz Caro, José Javier Anaya, Stéphanie Cazaux

**Affiliations:** 1 Faculty of Aerospace Engineering, Delft University of Technology, 2629 HS Delft, The Netherlands; M.P.E.Mendonck@student.tudelft.nl (M.M.); S.M.Cazaux@tudelft.nl (S.C.); 2 Centro de Astrobiología (CSIC-INTA), Ctra. de Ajalvir, km 4, Torrejón de Ardoz, 28850 Madrid, Spain; cgonzalez@cab.inta-csic.es (C.G.D.); munozcg@cab.inta-csic.es (G.M.M.C.); 3 Instituto de Tecnologías Físicas y de la Información Leonardo Torres Quevedo, (ITEFI-CSIC), c/Serrano 144, 28006 Madrid, Spain; m.g.hernandez@csic.es (M.G.H.); jj.anaya@csic.es (J.J.A.)

**Keywords:** ultrasonic techniques, water ice, liquid water, porosity, bubbles, wave propagation

## Abstract

This work studies ultrasonic propagation in liquid and ice water drops. The effect of porosity on attenuation of ultrasonic waves in the drops is also explored. The motivation of this research was the possible application of ultrasonic techniques to the study of interstellar and cometary ice analogs. These ice analogs, made by vapor deposition onto a cold substrate at 10 K, can display high porosity values up to 40%. We found that the ultrasonic pulse was fully attenuated in such ice, and decided to grow ice samples by freezing a liquid drop. Several experiments were performed using liquid or frozen water drops with and without pores. An ultrasonic pulse was transmitted through each drop and measured. This method served to estimate the ultrasonic velocity of each drop by measuring drop size and time-of-flight of ultrasonic transmission. Propagation of ultrasonic waves in these drops was also simulated numerically using the SimNDT program developed by the authors. After that, the ultrasonic velocity was related with the porosity using a micromechanical model. It was found that a low value of porosity in the ice is sufficient to attenuate the ultrasonic propagation. This explains the observed lack of transmission in porous astrophysical ice analogs.

## 1. Introduction

Comets are celestial objects formed at the outer edges of our solar system, i.e., sufficiently far away from the sun to preserve volatile species in the ice. This material originated in the local interstellar cloud and during the solar nebula epoch that evolved toward our current solar system. The ice mantle accretion process in space occurs by condensation of molecules onto the cold dust surfaces. At the lowest temperatures near 10 K, this accretion is expected to form highly porous ice, as occurs in laboratory simulations. This leads to a fluffy cometary ice structure since comets are made by agglomeration of icy dust particles [1,2]. The porosity values in such ice analogs measured in the laboratory can be up to 40% for H_2_O and CO_2_ ice [3,4] or up to 80% in water ice grown by vapor deposition at large incident angles [5].

This work explores the feasibility to study porosity by means of ultrasonic measurements in astrophysical ice analogs using a pulse–echo technique [6]. After several attempts to measure ultrasonic propagation in these analogs, we observed no signal in the Time-of-Flight (ToF) plots. We suspected that, as a consequence of the expectedly high porosity of the ice samples, the pulse was fully attenuated along the path from the transmitter to the surface of the ice film and its return to the receiver. Therefore, to gain insight in the influence of porosity on ultrasonic transmission, a number of experiments was performed with regular and sparkling water drops deposited directly on top of the transducer. In these experiments, the drop was first measured at room temperature and later frozen inside a freezer at −18 °C. After the drop was frozen, the transducer was connected to the ultrasonic pulse generator and an oscilloscope to obtain the ultrasonic signals. The experimental results were compared to numerical simulations that reproduce the ultrasonic pulse for different values of porosity in the liquid or ice drops.

Ultrasonic techniques allow non-destructive and non-intrusive analysis of samples [7]. The ultrasonic signal parameters, i.e., reflection and transmission coefficients, velocity, attenuation and scattering, are used to infer the properties of the material under study and real-time monitoring of the processes, e.g., [8,9]. This technique has been extensively employed for ice characterization. For instance, Antarctic ice cores provide useful information on climate records [10] and sea ice properties have been studied, e.g., [11]. In the laboratory, ice samples processes were monitored under controlled conditions, e.g., [12]. More related to our work is the study of different types of ice drops using ultrasonic techniques, which is of interest for aircraft icing phenomena, e.g., [13,14,15]. In previous works, we used ultrasonic pulse–echo inspection and modelling to study porosity in other materials [16], but there is to our knowledge no dedicated study of the porosity in ice using this technique.

## 2. Materials and Methods

The drop experiments and numerical simulations are described below.

### 2.1. Experiments on Frozen Drops

The top surface of a 10 MHz frequency transducer was covered with water. Two series of experiments were carried out. First, the transducer was covered by water (ultrapure water of Type 1 (Milli-Q, Merck Fisher Scientific, Darmstadt, Germany) and allowed to freeze, forming compact ice drops. Second, sparkling water containing CO_2_ bubbles was used to grow ice drops with porosity. Ultrasonic propagation was monitored without and with porosity in the liquid and ice water drops. A liquid ethanol drop experiment was performed to study the effect of a different liquid composition. The CO_2_ gas content of the bottle containing sparkling water was estimated as follows. The sparkling water bottle was weighed before opening. After the experiments it was filled with regular water and weighed again. The difference in weight was converted to the number of CO_2_ moles, which was 2.92 mol, and a density of 1.024 g cm^−3^ was obtained (2.3% higher than regular water). This value for the density falls in the range of sparkling water with CO_2_, i.e., a 2–3% increase. We refer to the highly cited work of J.E. García for details on this topic [17]. Because this amount of CO_2_ in the sparkling water samples is near 10.2% of the total volume, the average porosity of the drops should be similar to this value.

**Figure 1:**
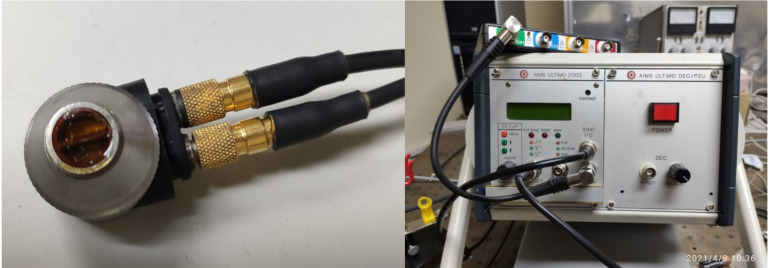
Ultrasonic transducer (**left**) and equipment (**right**) used for the experiments. The properties of the ultrasonic pulses (intensity and frequency) that arrive at the interface between the transducer surface and the drop samples depend on the settings of the ultrasonic pulser, the oscilloscope and the transducer characteristics.

The equipment used for the emission, amplification and reception of ultrasonic signals was the AIMS pulser/receiver. A Panametrics DHC713-R (Olympus, Waltham, MA, USA) dual element transducer with nominal frequency of 10 MHz, 66% bandwidth with 6 mm diameter was used. The two piezoelectric ceramic elements of this transducer act as emitter and receiver. It was excited with a voltage spike of 200 V. The received signal was amplified by 30 dB and a pulse envelope detector was applied. This transducer was selected taking into consideration the sample dimensions (drop size) and to avoid the interface pulse between the transducer and the drop overlapping with the pulse echo background. A four-channel Handyscope HS6 oscilloscope (TiePie, Sneek, The Netherlands) was used to digitize the ultrasonic signals with a sample rate of 200 MHz and a 12 bit resolution, see Figure 1.

The ice is grown inside a freezer for around 20 min. After the drop is frozen, the transducer was connected to the ultrasonic pulser and an oscilloscope to obtain the ultrasonic signals. Amplitude-scans (A-scans) were measured in this way by emitting a single ultrasonic pulse that was typically attenuated as it propagated along the drop, reaching the drop surface and bouncing back to the transducer, thus causing attenuation of the ultrasonic signal that can be measured. This is commonly known as the pulse–echo technique. The propagation time of the signal, known as time of flight (ToF) or pulse–echo time is represented in the *x*-axis and the intensity of the signal is provided in the *y*-axis of the A-scans.

### 2.2. Theoretical Study: Simulations of Wave Propagation and Influence of Porosity on Ultrasonic Velocity

In order to better understand the wave propagations in compact and porous water and ice, several simulations were made with in-house SimNDT software [18,19]. The mathematical foundation of SimNDT is a core based on the Elastodynamic Finite Integration Technique (EFIT) (P/SV: 2nd spatial-time order) for Elastic/Viscoelastic media to model the wave propagation in 2D for viscoelastic and elastic materials [16]. To improve the performance of this technique, the software was implemented using the standard for parallel programming of heterogeneous systems, Open Computing Language (OpenCL) [20]. The OpenCL framework provides an open, royalty-free and neutral-vendor parallel programming solution. It delivers a high degree of portability across different computing devices such as multi-core CPU’s and GPU’s. To simplify our OpenCL EFIT implementation, we used an open-source toolkit, PyOpenCL [21]. The PyOpenCL toolkit offers an attractive solution to implement parallel numerical codes using the high-level programming environment of Python, which requires much less explicit code to define and control the OpenCL programming model. A detailed description of the SimNDT core can be found in [18,19]. In addition, it is possible to generate a material with microstructure that is represented by a biphasic or three-phase model. The software incorporates a library with the elastic properties and density of several materials and allows the definition of new materials and/or the creation of another library. Depending on the selected inspection method, the configuration of the emitter/receiver, frequency, amplitude and wave type can be configured. Different boundary conditions can also be selected as well as their thickness. The aim of this set of simulations is to verify and justify the existence and origin of certain features that were observed in the response signal of the ultrasonic pulse.

**Figure 2:**
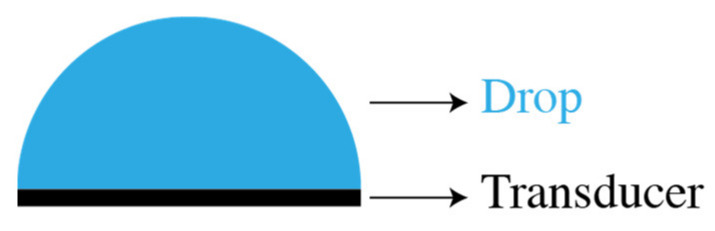
Simulated configuration for ultrasonic propagation in a drop.

The inspection setup consists of one transducer (6 mm in diameter) operating in pulse–echo mode and drives a raised cosine signal at 10 MHz, Figure 2. The ultrasonic waves are propagated in a drop of 3 mm radius filled with either liquid water or ice.

**Figure 3:**
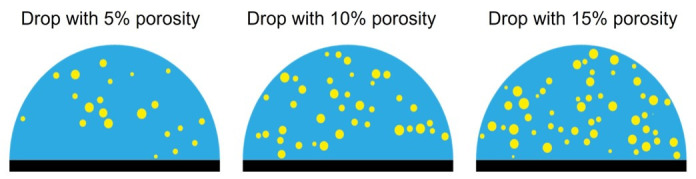
Drops with different values of porosity. Water and pores are represented in blue and yellow, respectively.

A set of simulations was made consisting of a liquid water or ice drop without and with pores, see Figure 3 for the pore distributions in the porous drops. The simulations of wave propagation in a drop were made to represent the conditions in the experiments. In the case of a drop with pores, these pores were considered to be full of air. It should be noted that the results obtained with our 2D model might differ from the experimental results that deal with real 3D drops. In particular, the porosity values used as input in this 2D model cannot be directly extrapolated to the porosity in the real drops. It will be shown that the large attenuation of the ultrasonic pulse calculated by the model corresponds in the experiments to larger porosity values. Despite this limitation, this model provides a useful representation of the interaction of the pulse with the drop surface and pores, which aids in the interpretation of the experiments. In the absence of pores, i.e., compact liquid/ice drops, we will show that this model reproduces very well the experimental ToF values.

The relationship between the porosity and the ultrasonic velocity in ices was also studied using a micromechanical model [22]. The micromechanical model deals with the effective elastic properties of composite materials as a function of microstructural properties that undergo different phase changes. These properties are related to the size, distribution and orientation of pores.

This micromechanical biphasic model considers that the sample material consists of two phases: solid matrix and pores. Assuming that the ice sample is an isotropic material, an equivalent isotropic medium is defined. From the expression of elastic constants tensor (*C*) presented in [22], this tensor is reduced to two independent elastic constants. The independent elastic constants *C*_11_ and *C*_44_, in reduced notation, obtained by symbolic notation introduced by Hill [23], can be calculated as:

(1) $$C_{11}=C_{11}^{m}+\frac{\nu^{i}\left[C_{11}^{i}-C_{11}^{m}-\frac{4}{3}\left(C_{44}^{i}-C_{44}^{m}\right)\right]\left(\langle T_{1111}\rangle +2\langle T_{1122}\rangle \right)}{\nu^{m}+\nu^{i}\left(\langle T_{1111}\rangle +2\langle T_{1122}\rangle \right)}+\frac{\frac{8}{3}\nu^{i}\left(C_{44}^{i}-C_{44}^{m}\right)\langle T_{1212}\rangle }{\nu^{m}+2\nu^{i}\langle T_{1212}\rangle }$$

(2) $$C_{44}=C_{44}^{m}+\frac{2\cdot \nu^{i}\left(C_{44}^{i}-C_{44}^{m}\right)\langle T_{1212}\rangle }{v^{m}+2\cdot v^{i}\langle T_{1212}\rangle }$$

where the superscripts *m* and *i* refer to matrix and inclusions, respectively, and *ν* represents the volume fraction of the phases. In this case, the inclusions are the pores, which can be full of air or carbon dioxide. *T_ijkl_* are the components of Wu’s tensor [24], which considers the geometry, distribution and orientation of pores. The geometry of pores is modelled as spheroids characterized by their aspect ratio [22].

The components of the elastic constant’s tensor of the composite (liquid water/ice with bubbles in our case) are related to the effective longitudinal (*V_l___eff_*) and shear (*V_t_eff_*) velocities as:

(3) $$V_{l\_eff}=\sqrt{\frac{C_{11}}{\rho_{eff}}}\;V_{t\_eff}=\sqrt{\frac{C_{44}}{\rho_{eff}}}$$

(4) $$\rho_{eff}=\rho^{m}\nu^{m}+\rho^{i}\nu^{i}$$

where *ρ_eff_* is the density of the composite and *ρ^m^* and *ρ^i^* are the densities of matrix and pores, respectively.

In this study of the influence of porosity in ice, the ice is considered to be the matrix, and the pores are represented as air inclusions with spherical geometry distributed randomly in the matrix [22]. The elastic constants of air are zero, those of the ice matrix are given in Table 1.

## 3. Results

In this section, the results obtained in the drop experiments and the numerical simulations are shown.

### 3.1. Drop Experiments

The compact liquid/ice drops were made with regular Milli-Q water while porous ice drops were made using sparkling water, see Section 2. An ethanol drop experiment was performed to study the effect of a different composition. A total of 52 drop experiments were performed to check the reproducibility of the results.

#### 3.1.1. Compact Liquid/Ice Drops

**Figure 4:**
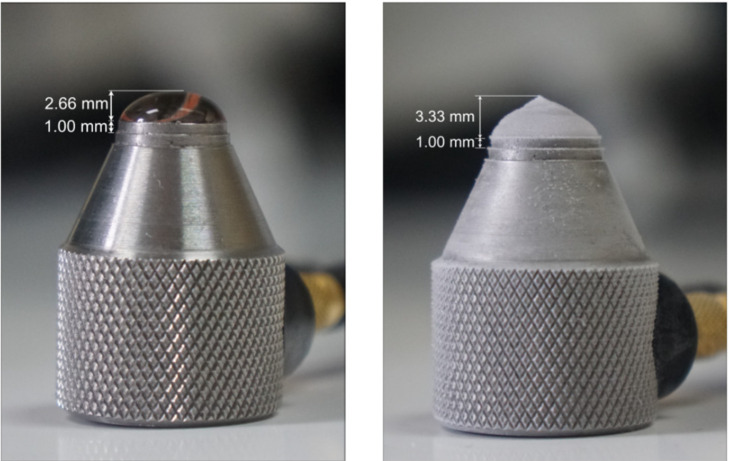
Optical determination of drop height from a liquid drop (**left**) and from a frozen drop (**right**). From these photographs, the well-known dimensions of the transducer element were used to estimate the drop dimensions in all the experiments.

For the determination of ultrasonic velocity, the drop dimensions need to be measured. They were estimated from the photographs. In each experiment the drop, liquid and ice, was photographed and from the dimensions of the transducers the dimensions of the drop were carefully estimated, an example is provided in Figure 4.

**Figure 5:**
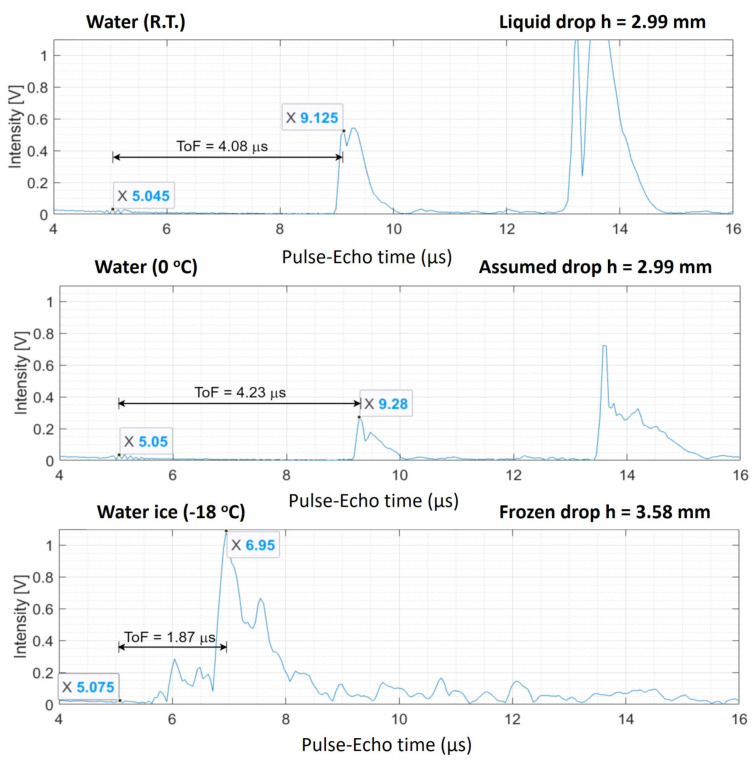
Typical set of A-scans for compact drop experiment. For three different temperature values, the ToF between the first arriving longitudinal reflected wave fronts from the top of the drop surface was subtracted and displayed in the respective graphs. This value was used to calculate the ultrasonic velocity in the drops by dividing the drop height.

The average drop height was 2.11 mm for liquid water and 3.33 mm for ice drops. When the drop was put inside the freezer, it cooled down from room temperature (R.T.) to freezing temperatures. The height of the drop is assumed to stay constant for its liquid phase (between R.T. and 0 °C). From the received ultrasonic signal, the peak that corresponds to the top of drop surface was identified, see Figure 5. The larger peak that appears near 14 µs is due to a second reflection of the wave front on the top of the drop surface; this effect will be explained in Section 3.1.3. The A-scan figures show the intensity of the ultrasonic response as a function of the ToF. The A-scans that refer to water at 0 °C correspond to drops in the freezer at the moment before they froze, this is before the ultrasonic velocity increased during the phase change, leading to a rapid displacement of the main peak toward shorter ToF values. This peak corresponds to bouncing of the ultrasonic pulse on the top of the drop surface and returning to the receiver. The lowest temperature achieved in the freezer was −18 °C. At this temperature the transducer continued working and the ultrasonic data was collected as usual.

The ToF related to the height of the drop was subtracted from the ultrasonic data by the time difference between the surface of the transducer signal and the first peak that was observed. Generally, the peaks generated by compact ice are steep and easy to spot, making the determination of the ToF for this type of drop straight forward. The ToF was determined from the maximum of the first peak. Figure 5 shows the A-scans for a typical experiment with compact ice. Because of the large difference in the ultrasonic velocity in liquid water and ice, the peak shifts close to the (relatively static) bump of the surface of the transducer when water freezes. The representation in the top panel of Figure 5 of the liquid water drop at R.T. displays a peak near 9 µs. This is the first reflection coming from the top of the drop. The second peak, at roughly 13 µs is a second reflection. It is larger than the first one as a consequence of the semi-spherical shape of the drop and the fact that the transmitting and receiving parts in the transducer are separated. This provokes multiple reflections creating positive interferences increasing the signal intensity. The ultrasonic velocity at different temperatures is calculated from the ToF in Figure 5 and the drop height, *h*, as follows:

(5) $$V_{R.T.}=\frac{2h}{\mathrm{ToF}}=\frac{2\times 0.00299\;m}{4.08\;\mu s}=1465.69\;m/s$$

(6) $$V_{0\;{}^{\circ}C}=\frac{2h}{\mathrm{ToF}}=\frac{2\times 0.00299\;m}{4.23\;\mu s}=1413.71\;m/s$$

(7) $$V_{-18\;{}^{\circ}C}=\frac{2h}{\mathrm{ToF}}=\frac{2\times 0.00358\;m}{1.87\;\mu s}=3828.88\;m/s$$

The deviations of these velocity values with those published in other works for water films are less than 2% for the three temperatures, see Table 2.

##### Statistical Results for Compact Liquid/Ice Water Drops

In total, 22 out of the 52 drop experiments were performed with compact ice. Not all of them had readable ToF’s and in some of them, visible cracks in the ice made the ultrasonic signal disappear. The useful experiments were used for the statistics reported in Table 3.

The results in Table 3 illustrate the need for a large dataset of drop experiments. Even though the standard deviation can be up to 7.05%, the difference between the mean of the drop experiments at R.T. and the literature value is only 0.23%. The larger difference with respect to the literature value in the ultrasonic velocity measured in water at 0 °C could have been caused by the fact that the drop temperature could not be measured directly, and the height could be smaller compared to the R.T. height of the drop. Nonetheless, the performance of this experimental testing procedure has a satisfying accuracy since the drop geometry can vary between experiments, in particular in the frozen drops, leading to higher deviations from the literature values. The similarity of the ultrasonic velocity values measured in the liquid/ice drops with those reported in the literature for liquid water and ice samples of known thickness served to validate the experimental protocol. The set of drop experiments dedicated to study the effect of porosity on ultrasonic propagation are presented in the next section.

#### 3.1.2. Porous Liquid/Ice Drops

To make water ice porous under atmospheric conditions, sparkling water was used. The molar mass of CO_2_ in the sparkling water sample corresponds to a gas volume of 10.2% of the total volume (water + CO_2_), see Section 2. The bubbles created by the sparkling water in the drop increase the attenuation of the ultrasonic signal very significantly. This makes it harder to determine which peak from the ultrasonic A-scans comes from the top of the drop surface. The procedure used to identify such peak is presented by means of two experiments that were selected to emphasize the largest differences that may appear between experiments.

##### Drop Experiment 1 Using Sparkling Water

**Figure 6:**
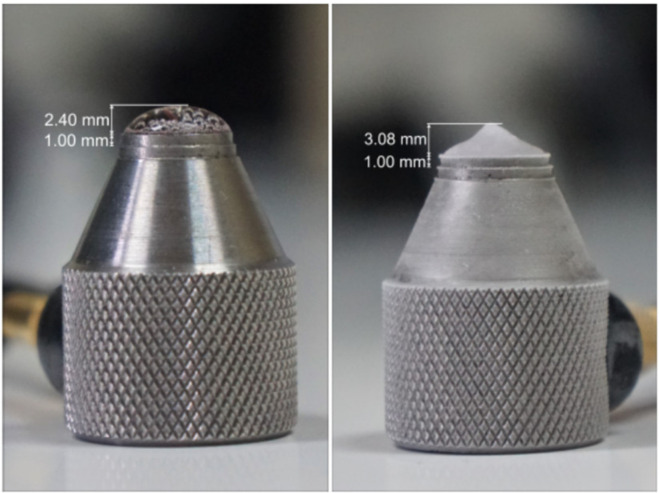
Optical determination of drop height at room temperature (**left** panel), or as a frozen drop (**right** panel). These pictures correspond to Ice Drop Experiment 1, discussed in the body of the article.

For this type of experiments, the determination of the drop height is done in the same manner as for the drops with compact ice. The left and right images of Figure 6 show the height measurement for the liquid and the frozen drop. While comparing the ice drop in the image on the right to the image of compact ice on the right in Figure 4, the difference in the shape of the frozen drop stands out. While the drop for compact ice had rounder edges in general, the frozen drop for porous ice often tends more towards a conical shape. This is immediately correlated to the path the ultrasonic wave will travel. In addition, the ultrasonic wave will be scattered by the bubbles.

**Figure 7:**
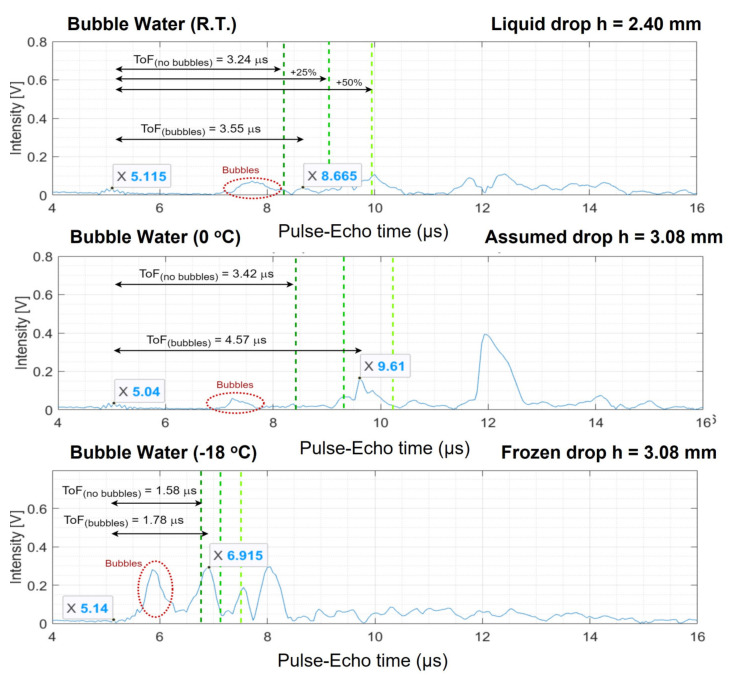
Typical set of A-scans for Drop Experiment 1. For three different temperature values, the ToF of the hypothetical compact ice (without bubbles) is drawn as the dark green dotted line. The other green dotted lines indicate lines of 25% and 50% beyond this ToF. The ToF of the top of the porous drop is labelled as ToF_(bubbles)_. The peaks that likely indicate bubbles are shown in the red dotted circles.

From the ultrasonic data, the A-scans were analysed. The reflected signals exhibit peaks that look different compared to the compact ice drops with no bubbles. The intensity of the peaks dropped and the amplitude of the noise and intermediate reflections that originate from the bubbles increased. In the case of the experiments with compact water, the top of the drop surface can be identified as the first peak that appears in these plots, but when comparing the ToF of the first peak in the experiments with bubbles to the one expected from the literature, one could see that the first peak that appears is usually located before the literature ToF value, see Figure 7.

For Drop Experiment 1, the procedure of defining the top of the water and ice drops is clarified by the graphs in Figure 7. Every A-scan is shown as two boxes where the bump from the transducer surface and the peak representing the top of the drop surface are reported. The left one comes from the surface between the transducer and the liquid water or ice. The other one is the estimated peak that resembles the top of the drop. The expected ToF for compact ice (ToF_(no−bubbles)_) is shown as the dark green dashed line in the graph. The effective ultrasonic velocity in porous water or ice is slower causing the peak corresponding to reflection on the top of the drop surface to appear behind the green dashed line. This is why any peak on the left of the green dotted boundary can be considered a consequence of the bubbles that are present in the drop. The signature of the bubbles is pointed at by the red circle in these A-scans. The two lighter green dashed lines are the positions in the graph for which the theoretical literature ToF of a compact drop is 25% and 50% more. Following the analogy applied on the compact ice drops in Equations (5)–(7), the ultrasonic velocity for the drop can be found. This results in V_R.T._ = 1352.11 m/s, V_0 °C_ = 1347.35 m/s and V_−18 °C_ = 3468.96 m/s for the two liquid water drops with different temperatures and the ice drop. This corresponds to 91.30%, 96.03% and 89.21% of the ultrasonic velocity with respect to water without bubbles at R.T., at 0 °C and to ice at −18 °C, respectively.

##### Drop Experiment 2, also Using Sparkling Water

**Figure 8:**
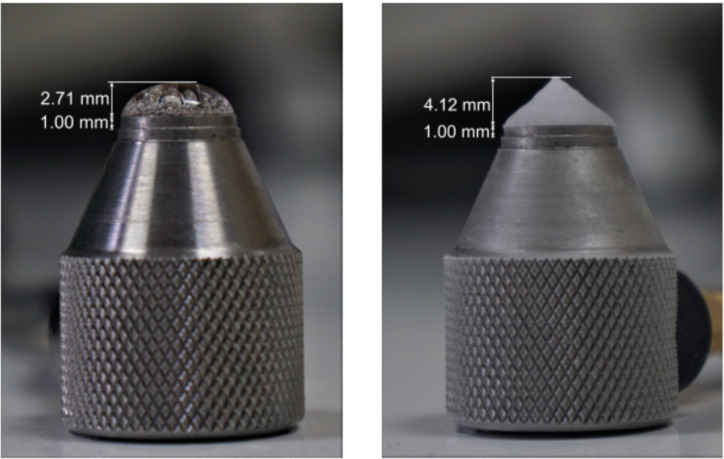
Optical determination of drop height at room temperature (**left** panel), or as a frozen drop (**right** panel). These pictures correspond to Ice Drop Experiment 2, discussed in the body of the article.

**Figure 9:**
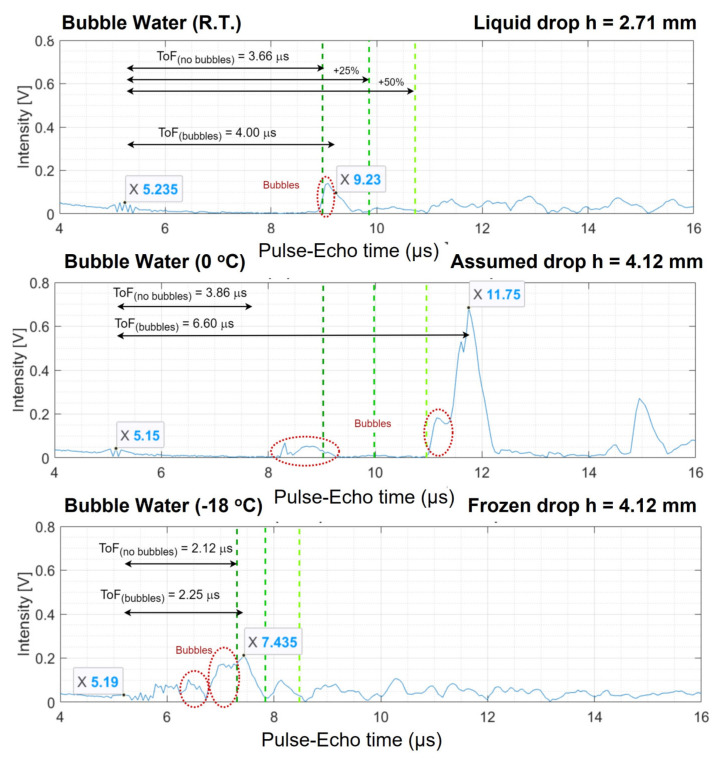
Typical set of A-scans for Drop Experiment 2 for three different temperature values, the ToF of the hypothetical compact ice (without bubbles) is drawn as the dark green dotted line. The other green dotted lines indicate lines of 25% and 50% beyond this ToF. The ToF of the top of the porous drop surface is labelled as ToF_(bubbles)_. The peaks that likely indicate bubbles are shown in the red dotted circles.

As mentioned above, a second porous ice drop experiment is discussed to highlight differences between A-scans. For the compact ice drop experiments, all experiments showed a similar and predictable behaviour in their measured ultrasonic response, the porous ice drop experiments showed less consistency and higher unpredictability. Similar to the previously discussed experiments, the determination of the drop height can be seen in Figure 8 and the set of A-scans are presented in Figure 9. The graphs in Figure 9 are provided with marks on the points of interest and corresponding ToFs. The suspected signatures of bubbles in the ultrasound is marked with red circles. When comparing the A-scan at room temperature with the one at 0 °C, one can see that a new bubble has grown during the cooling of the drop while the reflected signal moves to the right with respect to the expected position obtained from literature values for compact drops [25] (the green vertical lines). It could be argued that Drop Experiment 1 also displayed a new shoulder in the peak around 9.5 µs, see Figure 7, but it was not considered as a new bubble because the difference in ToF between the peak and the shoulder is very small (about 0.25 µs) and could thus be an integral part of the main peak. It could be caused by the reflection of the first bubble that appears as a superposition on the slope of that peak. On the other hand, the shoulder in Drop Experiment 2 was considered to be a bubble because the ToF from the main peak is larger (about 0.75 µs) and therefore more distinct. In fact, the shoulder in Drop Experiment 2 has a twofold higher intensity than the first bubble. Therefore, even considering that the superposition of the reflection of the first bubble would be present there, there must be something different.

This indicates that the drop would have grown in the freezer and the height measurement of the liquid drop in Figure 8 is no longer valid. The determination of the height of the ice drop was found to be a better estimate of the real height of the drop at 0 °C. Therefore, the measurement performance of the liquid drop at freezing temperature should be considered lower than the other ones. Selecting which peak from the ToF corresponds to the top of the drop surface was a difficult procedure in this experiment. The freedom of the bubbles in the water drops cause the ultrasonic peaks to change in intensity and ToF over time, which is why a single A-scan frame cannot provide enough information to derive the ToF of the height of the drop. The ones selected in the graphs were found to be the best estimates after analysing how peaks propagate over time for the entire duration of the experiment. After determination of the drop height and its corresponding ToF, the estimated ultrasonic velocity can be found. This results in V_R.T._ = 1358.84 m/s, V_0 °C_ = 1247.47 m/s and V_−18 °C_ = 3667.41 m/s for the two liquid water drops with different temperatures and the ice drop, respectively. This corresponds to 91.75%, 88.91% and 94.31% of the ultrasonic velocity with respect to water without bubbles at R.T., 0 °C and ice at −18 °C, respectively.

###### Statistical Results for Porous Liquid/Ice Water Drops

The useful experiments performed with porous liquid/ice drops and the statistical outcome are summarized in Table 4. The mean values with their standard deviations are shown in the table from which can be seen that the average ultrasonic velocity for these experiments is slightly lower than the literature values for compact water.

#### 3.1.3. Ethanol Drops

**Figure 10:**
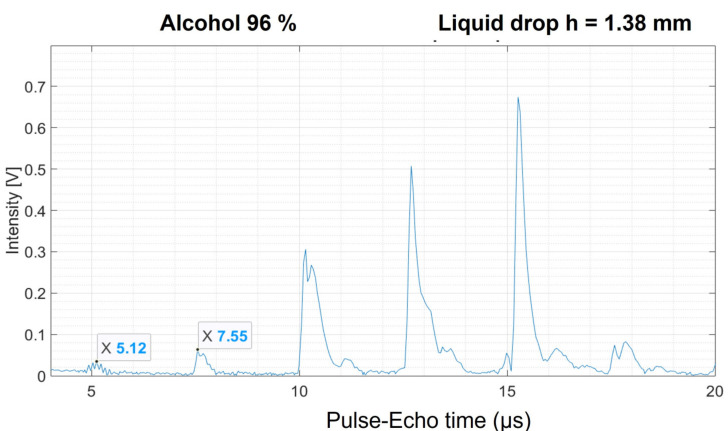
Room temperature A-scan of a drop of ethanol with 96% purity. The series of peaks with increasing intensity is the result of resonances created by the metallic exterior of the transducer.

To benchmark the technique on substances other than water, ethanol was used with a purity of 96%. From Figure 10 it is clear that the ultrasonic propagation in the drop does not encounter a high degree of damping. The first peak after the peak of the surface (at 7.55 µs) of the transducer can be attributed to the top of the drop surface.

From the ultrasonic ToF of plot in Figure 10, an ultrasonic velocity of 1131.69 m/s can be derived. The literature value for the ultrasonic velocity of pure ethanol is found to be 1142 m/s [26], which differs only by 0.90% from our value. Other works report higher values, about 1182 m/s or even close to 1300 m/s [27,28]. As a sidenote, it should be added that during this experiment, slowly and steadily, the ToF of the peaks became increasingly shorter (peaks moved to the left). This is most likely related to evaporation of the ethanol at room temperature. Therefore, the height of the drop should be monitored frequently when experiments require a long exposure to atmospheric conditions. The most significant observation in this ethanol experiment, which also occurred to a lesser extent in the liquid water drops, is the increase of the subsequent peak intensities due to new reflections of the wave front on the top of the drop surface. This peculiar effect is due to resonances created by the metallic exterior of the transducer. When the transducer is seen from above, this metal appears as a “ring” around the transducer surface and creates such resonances. Indeed, if a small water drop is placed in the middle of the transducer surface there is no direct contact with the metal and resonances do not occur. We decided however to grow large drops covering the whole transducer surface for better reproducibility of the experiments and a more accurate estimation of the drop dimensions. Then, to discard the effect of these resonances that appear in the liquid drops, we estimated the ToF considering only the first peak signal, this is the one corresponding to the first reflection of the wave front on the top of the drop surface.

### 3.2. Numerical Simulation of Propagation Waves in Liquid Water and Ice Drops

#### 3.2.1. Liquid Water and Compact Ice Drops

**Figure 11:**
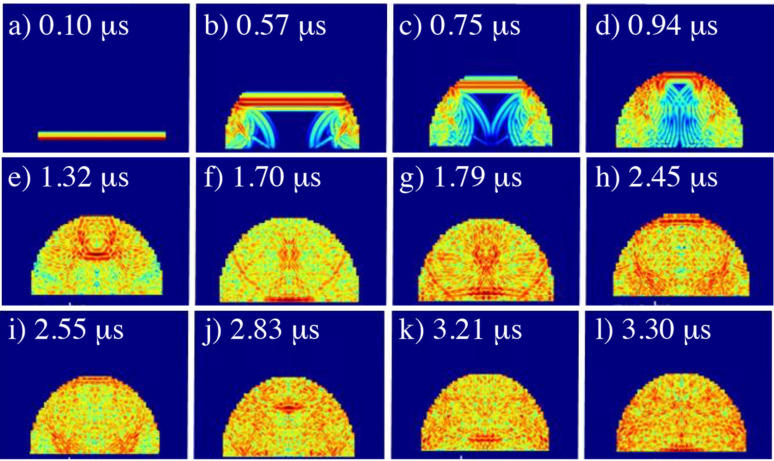
Simulated wave propagation in a 3 mm ice drop. The transducer that sends and receives the ultrasonic signal is located on the flat surface at the bottom of the panels. The amplitude of the ultrasonic waves is represented by the red and yellow colours. Darker red and yellow represent stronger waves (higher amplitudes), while blue colours correspond to low amplitudes.

The average height for the ice drops from the experiments was 2.96 mm. Figure 11 shows a set of relevant frames from the simulation of ultrasonic waves in a 3 mm ice drop. Furthermore, the width of the drop was also set to match the one of the transducers used in the experiments and the geometry was predefined to resemble a drop. The simulation showed that the shape indeed has a significant influence on the acoustic wave propagation. From the top left to the bottom right, the amplitude of the waves is represented by red and yellow colours. The wave propagates from top to bottom in the frames and are reflected when they interact with a surface. A description of the chronological frames follows:

a. At 0.10 µs, a longitudinal ultrasonic pulse is transmitted from the transducer and the wave front penetrates into the ice drop.
b. At 0.57 µs, the wavefront encounters the curvature of the drop surface after which the longitudinal wave is reflected. These reflections contribute to a horizontal propagation within the drop. The undisturbed part of the longitudinal wave continues to propagate towards the top of the drop surface.
c. At 0.75 µs, the first horizontal reflected wavefronts meet in the centre of the drop and start interfering with each other.
d. At 0.94 µs, the undisturbed wavefront of the original pulse reaches the surface at the top of the drop. Meanwhile, the horizontal wavefronts from the reflections dominate the reading of the transducer. This is the reason why reflective signals are observed in the experiments before the reflection peak coming from the top of the drop.
e. At 1.32 µs, the reflection from the top of the drop is travelling towards the transducer.
f. At 1.70 µs, impact of the reflection of the ultrasonic signal from the top of the drop onto the transducer. This impact creates the first large peak in in the measured ultrasonic response.
g. At 1.79 µs, after the impact, this strong signal will be reflected back to the top of the drop.
h. At 2.45 µs, the reflected signal approaches the surface of the top of the drop again. Once more, disturbing interactions of other pressure waves interact with the transducer.
i At 2.55 µs, after this second impact it can be seen that the reflected signal is significantly attenuated by this impact.
j. At 2.83 µs, interference of a curved wave front moving towards a common centre point makes the amplitude locally higher, resulting in a dark-red dot in the simulation frame moving towards the transducer now.
k. At 3.21 µs, close to the transducer the wave front is more dispersed, which decreases its intensity.
l. At 3.30 µs, the second impact on the transducer of the reflected wave from the top of the drop. This creates a second, smaller peak.

**Figure 12:**
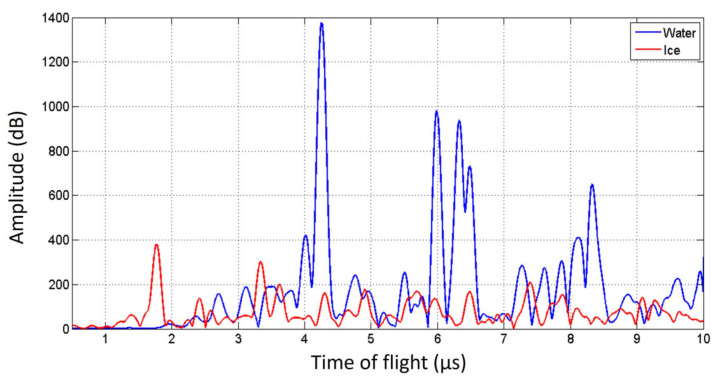
A-scans in a 3 mm liquid water (**blue trace**) and ice drop (**red trace**). The ice data corresponds to the snapshots presented in Figure 11.

This simulation entails the ultrasonic reading as seen from the transducer as well, which is presented in Figure 12. The time that each frame from Figure 11 represents can be related to the *x*-axis of this graph. For comparison, the simulation corresponding to a liquid water drop of the same size is shown in the left panel of this figure. Right after 0 µs, the longitudinal pressure wave sent by the transducer can be seen. As explained above, the first large peak at 1.70 µs can be attributed to the top of the drop surface. The second large peak at 3.30 µs is a reflection of the first large peak that came from the top of the drop. In between and in front of the first peak, acoustic disturbances are present as a result of the drop geometry. It is important to clarify that this simulates a wave front that covers the entire bottom of the drop. In the experiments the receiver is half of the surface of the transducer. This would make the propagation within the drop look more chaotic because the propagation of the longitudinal waves will have more horizontal movements, but the ToF of the large peak would not change significantly.

The ToF values in these simulations agree very well with those measured in the liquid and ice compact water drop experiments presented in Figure 5.

#### 3.2.2. Porous Liquid and Ice Drops

**Figure 13:**
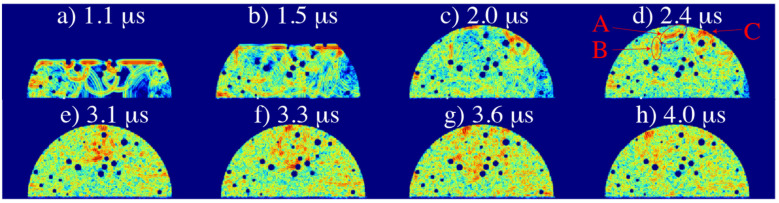
Simulated wave propagation in a 3 mm liquid water drop with 5% porosity. The transducer that sends and receives the ultrasonic signal is located on the flat surface at the bottom of the panels. The time for each snapshot after the start of the pulse is indicated. Darker red and yellow represent stronger waves (higher amplitudes), while blue colours correspond to low amplitudes. Although the dissipating effects caused by pores have a strong impact on wave front propagation, a small fraction of the wave front is reflected by the top of the drop surface after 2 µs and arrives back to the transducer after 4 µs. At 2.4 µs, the reflected wave front A is about to merge with two intense wave fronts; on the left, B, and on the right side, C.

A similar set of numerical simulations were performed for porous liquid and ice drops to understand the influence of porosity in the sparkling water experiments. The simulation creates a cross section of the drop with randomly filled porosity bubbles that have random radii between 0.05–0.15 mm. The drops were filled with bubbles until a certain percentage of surface area was covered by these bubbles. Figure 13 has a porosity of 5% for a liquid water drop. It shows that while the wave front of the pulse propagates through the drop, the bubbles act as dispersive elements in the drop. By the time the wave front reaches the top of the drop surface, it has already decreased significantly in intensity. It decreases even further since the wave has to travel back to the flat transducer surface. A description of this figure follows:

a. At 1.1 µs, the wave front is dispersed by pores near the transducer forming numerous peaks in the A-scan.
b. At 1.5 µs, the wave front is thus considerably attenuated by the pore interactions on its way to the drop surface.
c. At 2.0 µs, arrival at the top of drop surface, the weakened front wave travels back in the direction of the transducer.
d. At 2.4 µs, the reflected wave front A is about to merge with two intense wave fronts; on the left, B, and on the right side, C. Wave front C was also reflected from the drop surface, while wave front B was a residual that did not reach the drop surface.
e. At 3.1 µs, the coalescence of these waves produces a reinforced new front wave.
f. At 3.3 µs, this new front wave interacts with the pores located around the center of the drop.
g. At 3.6 µs, the new front wave is heavily dispersed by the pores.
h. At 4.0 µs, the first fragment of the dispersed front wave arrives to the transducer.

**Figure 14:**
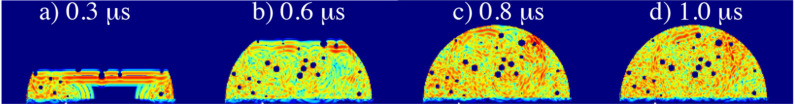
Simulated wave propagation in a 3 mm ice drop with 5% porosity. The transducer that sends and receives the ultrasonic signal is located on the flat surface at the bottom of the panels. The time for each snapshot after emission of the pulse is indicated. Darker red and yellow represent stronger waves (higher amplitudes), while blue colours correspond to low amplitudes. Even for this low value of porosity, the strong dissipating effects caused by pores do not allow the reflected wave front (reaching the top of the drop surface after 0.8 µs) to travel back to the transducer.

The simulation corresponding to the equivalent ice drop with 3 mm height and 5% porosity is presented in Figure 14 as described below:

a. At 0.3 µs the wave front is dispersed by pores near the transducer forming numerous peaks in the A-scan.
b. At 0.6 µs the wave front is considerably attenuated by the pore interactions on its way to the drop surface. Numerous high-intensity waves arrive continually to the transducer producing peaks in the A-scan. The intensity of the received signal is, however, highly variable.
c. At 0.8 µs the wave front arrives at the top of drop surface. The weakened wave front then travels back in the direction of the transducer.
d. At 1.0 µs, unlike the liquid drop case, there is no obvious merging of wave fronts and the reflected wave front does not find its way back to the transducer.

**Figure 15:**
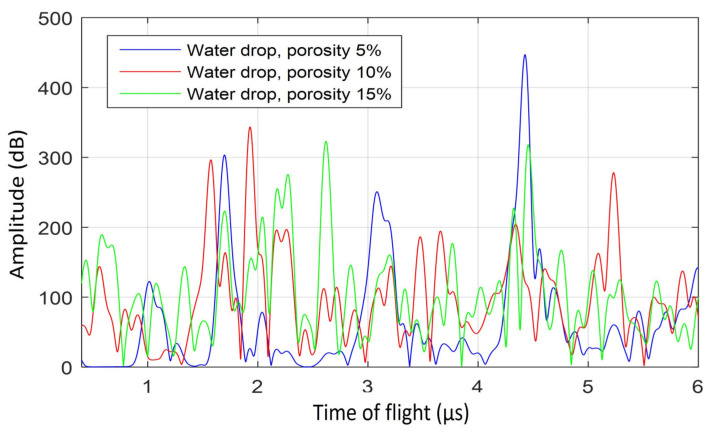
Simulated A-scans of the propagation in a 3 mm liquid water drop. The plots show the response of drops with 5%, 10% and 15% porosity in blue, red, and green, respectively.

**Figure 16:**
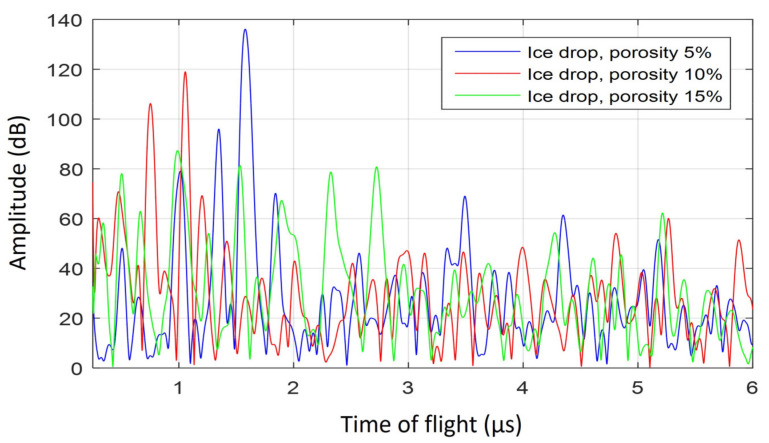
Simulated A-scans of the propagation in a 3 mm water ice drop. The plots show the response of drops with 5%, 10% and 15% porosity in blue, red, and green, respectively.

The plots in Figure 15 and Figure 16 show the simulated response in porous liquid water drops and porous ice drops, respectively. These figures show the response with three different porosity values, these are 5%, 10% and 15%. The snapshots corresponding to 5% porosity were presented in Figure 13 and Figure 14 for the liquid and ice drop, respectively. The simulated porosity leads to a high attenuation. As discussed in Section 2.2, the porosity in this 2D model would correspond to a higher porosity value in the real drops. As a consequence of this attenuation, the ultrasonic pulse barely reaches the top of the drop surface, and when it does, it may not propagate back to the receiver because bubbles continue to attenuate the reflected signal. The prominent peak associated with the reflection on the top of the drop surface is thus not observed, except in the liquid drop with 5% porosity, where the peak near 4.4 µs is related to reflection from the top of the drop surface. This ToF value of 4.4 µs is slightly delayed with respect to the experimental ones in Figure 5 for compact liquid drops at room temperature and 0 °C (top and middle panels). The smaller ultrasonic signatures that appear in Figure 15 and Figure 16 are due to reflections caused by the bubbles where the wave front does not make its way to the top of the drop surface.

## 4. Discussion

When comparing the results of experiments of compact drops with porous drops, it was observed that the average ultrasonic velocity in porous drops is lower than in compact drops. A summary of the results is reported in Table 5. The standard deviation (SD) is higher for porous drop velocity measurements. The difference between the average ultrasonic velocity for both drop types increases when the temperature decreases from 9.05% at room temperature to 12.4% for the frozen drop.

### Comparison of Theoretical and Measured Velocity from Micromechanical Model

**Figure 17:**
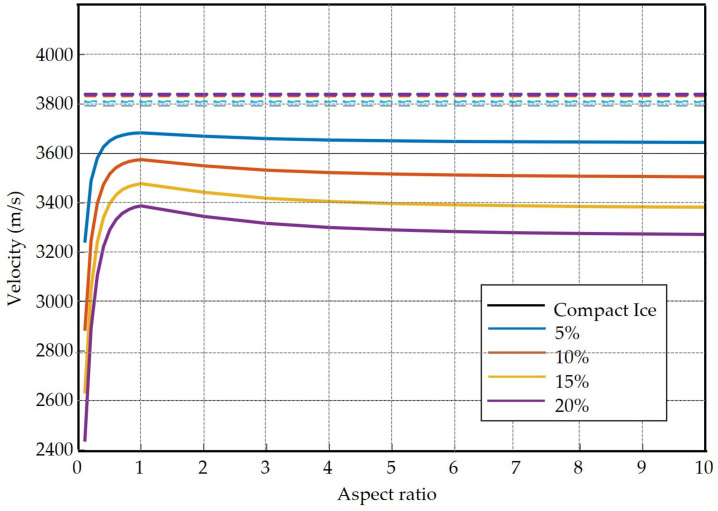
Comparison of theoretical and measured velocities of compact ice drop experiments.

**Figure 18:**
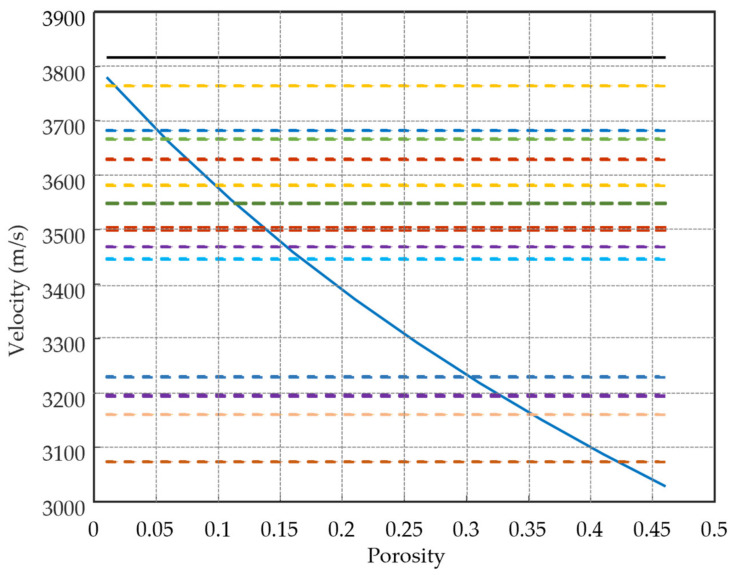
Comparison of theoretical (**solid lines**) and measured (**dashed lines**) ultrasonic velocities in porous ice drop experiments. The theoretical values were provided by the micromechanical model for increasing porosity. It can be observed that the velocities obtained in most experiments correspond to porosities in the 5% to 16% range. The expected average porosity of 10.2%, which corresponds to the gas-to-liquid volume in sparkling water, falls within these values. The four experiments at the bottom are anomalous, their low velocities are attributed to a poor contact of the ice drop with the transducer or cracks in the ice.

Figure 17 and Figure 18 show the comparison of theoretical and measured velocities of compact ice drop and porous ice drop experiments, respectively. The dashed line represents the results obtained in the experiments at the laboratory. Figure 17 shows the theoretical ultrasonic velocities depending on the porosity and the aspect ratio, i.e., the ratio of width to height of the pores. It verifies that the velocities of drop experiments are according to the velocity of ice (3807 m/s). They stand fairly well above those numerically simulated for porous ice drops. Figure 18 shows that from the comparison of velocities for a porous drop, it is possible to relate the ultrasonic velocity with the porosity present in the drops. Although there is a significant dispersion between the velocities measured in the set of porous drop experiments, most drops display values near 10 ± 5%. The 4 experiments with velocities lower than 3300 m/s could be explained by a poor contact of the ice drop with the transducer or cracks that appeared in the ice.

## 5. Conclusions

This work served to study in detail the propagation of ultrasonic waves in liquid and frozen waters drops, either compact or showing different values of porosity. An estimate of the ultrasonic velocity in various drop experiments could be made, showing the expected decrease in velocity in porous drops. The drop porosity in the experiments was expected to be around 10.2% in most cases, since this was the CO_2_ gas volume in the sparkling water sample used in our experiments. This is compatible with the results presented in Section 4, where the estimated velocities in most experiments correspond to porosities in the 5% to 16% range.

The numerical simulations allowed the study of wave propagation in the liquid/frozen water drops as a function of the time of flight, and for three porosity values of 5%, 10% and 15%. These simulations show that only for the case of 5% porosity in the liquid drop, an attenuated peak was measured corresponding to reflection from the top of the drop surface. Higher porosity values of 10% and 15% only displayed small peaks due to reflections of the bubbles since the wave front in these drops did not reach the top of the drop surface. This explains the lack of peak detection in our experiments with ice films grown from vapor deposition under vacuum, in order to reproduce fluffy cometary ice, with expected porosities up to 40%. We conclude that ultrasonic measurements can probably not be used for the study of such high porosity ices, but they might serve to detect a transition from porous to compact ice, which occurs during ice warmup. This issue deserves more research work. In particular, the hemispherical drop shape in our experiments causes reflection of the wave front to occur mainly along the symmetrical axis of the drop, and therefore pores along this axis lead to strong attenuation. This effect will not occur in flat ice films grown in vacuum chambers by vapor/gas deposition to mimic interstellar ice. Finally, ultrasonic measurements allowed the detection of bubbles in the drop experiments. This finding, in combination with the decrease in ultrasonic velocity measured in porous drops, are a direct consequence of porosity in liquid and frozen drops.

## Figures and Tables

**Table 1:** Elastic properties of the ice matrix.

	C_11_ (GPa)	C_44_ (GPa)	ρ (kg/m^3^)
Ice	13.33	3.7	920

**Table 2:** Ultrasonic velocity values of this particular experiment compared with literature values [25].

	V_R.T._	V_0__°C_	V_−18__°C_
Experimental (m/s)	1465.69	1413.71	3828.88
Literature (m/s)	1481.00	1403.00	3888.50
Difference (m/s)	15.31	−10.71	59.62
Difference (%)	1.03	−0.76	1.53

**Table 3:** Ultrasonic velocity values of all drop experiments compared with literature values [25]. The mean values and the standard deviations (SD) are included. Differences are given in m/s and in %.

	V_R.T._	V_0__°C_	V_−18__°C_
# of useful experiments	18	8	8
Literature (m/s)	1481.00	1403.00	3888.50
Mean experiment (m/s)	1477.55	1454.47	3922.25
SD Experiments (m/s)	104.18	62.20	151.28
SD Experiments (%)	7.05	4.28	3.86
Difference (m/s)	3.45	−51.47	−33.75
Difference (%)	0.23	−3.67	−0.87

**Table 4:** Ultrasonic velocity values of porous drop experiments compared with literature values of “compact” liquid water and ice with no bubbles [25]. The mean values and the standard deviations (SD) are included.

	V_R.T._	V_0__°C_	V_−18__°C_
# of useful experiments	13	20	19
Compact Lit. (m/s)	1481.00	1403.00	3888.50
Mean experiment (m/s)	1343.84	1299.97	3435.99
SD Experiments (m/s)	123.59	145.29	376.58
SD Experiments (%)	9.20	11.18	10.96
Difference (m/s)	137.16	103.03	452.51
Difference (%)	9.26	7.34	11.64

**Table 5:** Summary of statistical values of the ultrasonic velocity derived from the compact and the porous drop experiments.

		V_R.T._	V_0__°C_	V_−18__°C_
Compact ice drop	Mean (m/s)	1477.55	1454.47	3922.25
	SD (m/s)	104.18	62.20	151.28
	SD (%)	7.05	4.28	3.86
Porous ice drop	Mean (m/s)	1343.84	1299.97	3435.99
	SD (m/s)	123.59	145.29	376.58
	SD (%)	9.20	11.18	10.96
Difference	Mean (m/s)	133.71	154.5	486.26
	Mean (%)	9.05	10.62	12.40

## Data Availability

Not applicable.
